# Implementing proposed reforms of the Mental Health Act for people with intellectual disability and autism: the perspective of multidisciplinary professionals in intellectual disability teams

**DOI:** 10.1192/bjo.2022.604

**Published:** 2022-11-14

**Authors:** Bharat Velani, Indermeet Sawhney, Regi T. Alexander, Sophie Shardlow, Asif Zia

**Affiliations:** Adult Learning Disability Services, Hertfordshire Partnership University NHS Foundation Trust, UK; School of Life and Medical Sciences, University of Hertfordshire, UK; School of Life and Medical Sciences, University of West London, UK

**Keywords:** Autism spectrum disorders, human rights, intellectual disability, in-patient treatment, psychiatry and law

## Abstract

**Background:**

A recent government white paper sets out proposals for reforms to the Mental Health Act 1983 (MHA). Some of these proposals affect people with intellectual disabilities and/or autism.

**Aims:**

To explore both positive and unintended negative effects of the proposed reforms by gathering the perspectives of healthcare workers from multiple disciplines, working with intellectual disability and/or autism in community and in-patient settings.

**Method:**

A 14-question electronic questionnaire, comprising free-text, multiple choice and five-point Likert scale responses, was sent out via email between April and July 2021, to all multidisciplinary team professionals working in specialist intellectual disability community and in-patient teams in Hertfordshire Partnership University NHS Foundation Trust.

**Results:**

There were 45 responders, of whom 53% worked in in-patient settings and 47% in out-patient teams. Respondents comprised healthcare professionals from multiple disciplines, 80% of which were non-medical. Most responders agreed with the general principles of the proposed reforms. However, 80% felt there would be potentially unintended consequences, and 76% thought that substantial investment in community services was required in advance of the proposed reforms.

**Conclusions:**

The proposed MHA reforms may have unintended consequences for people with intellectual disabilities and/or autism. The findings of this study raised key concerns that need to be explored further and addressed before the MHA reforms are implemented. These include community provision, safeguards and use of the Mental Capacity Act, the potential for under or overdiagnosis of mental illness, and effects associated with the criminal justice system.

## Proposed Mental Health Act reforms

In January 2021, the Department of Health and Social Care published their long-awaited white paper proposing radical reforms to the Mental Health Act (MHA) 1983 (as amended in 2007) in England and Wales. After a process of consultation, a draft Mental Health Bill was published for pre-legislative scrutiny.^1^ This comes in response to Professor Sir Simon Wessely's independent review, published in 2018, which offered welcomed insights into current difficulties with the use of the MHA in England and Wales.^2^ Within the broad range of recommendations set out by the white paper are proposals for reform that will specifically affect people with intellectual disabilities and/or autism in England and Wales. It is these specific reforms that are the focus of this paper. Summarised below are some of the key features set out in the white paper:

- Intellectual disabilities and autism will no longer be considered mental disorders warranting compulsory detention under Section 3 of the MHA in England and Wales, a commonly used treatment order.
- The use of Section 2 of the MHA in England and Wales, a common assessment order allowing hospital detention for a 28-day period of assessment, is still permitted if there is a substantial risk of harm to themselves or others with a probable mental health cause. However, detention must end if underlying mental illness is not thought to be the cause of presentation.
- Changes apply to people with intellectual disabilities and/or autism under the MHA civil sections and not those in the criminal justice system.
- Care, Education and Treatment Reviews (CETRs) will be placed on a statutory footing.

The current MHA Codes of Practice state that, without evidence of underlying mental illness, having an intellectual disability alone does not warrant detention for treatment under Section 3 of the MHA. However, there is a caveat that allows detention for treatment under Section 3 if a person with an intellectual disability is presenting with associated challenging behaviour that poses a risk to themselves or others, and that represents ‘abnormally aggressive or seriously irresponsible conduct’.^3^ This is the case in the MHA Codes of Practice for both England and Wales.

## Cultural context and driving thematic factors for reform

There has long been a push from human rights and patient/carer advocacy groups to remove people with intellectual disabilities and autism from the scope of the MHA.^4,5^ The underlying aim is to promote autonomy and liberty for all people, as set out by the Human Rights Act 1998. It has been argued that current mental health legislation is not compatible with the United Nations Convention of Rights of Persons with Disabilities and directly violates key principles such as Article 12, which sets out that people who have a disability should have ‘equal recognition before the law’.^6^ The present wording of the MHA can be considered discriminatory because the caveat that allows for detention for treatment under Section 3 in the case of ‘abnormally aggressive or seriously irresponsible conduct’ only applies to people with intellectual disabilities.

In response to the abuse and neglect uncovered at Winterbourne View hospital, the government launched its ‘Transforming Care Programme’ in 2012, which aimed to reshape intellectual disability services to support discharges and prevent inappropriate admissions.^7^ The government also introduced CETRs, with the aim of reducing in-patient admissions and improving quality of care during admissions by helping to improve current and future care planning, including plans for leaving hospital. They are intended to occur before admission and involve a panel of independent people, including an expert by lived experience, a qualified clinical expert and the local commissioner in charge of paying for the patient's care. The panel asks key questions and makes recommendations that lead to improvements in safety, care and treatment.

The ‘Building the Right Support’ 2015 national plan for England set out to reduce the number of in-patients to between 1300 and 1700, a target that has not been met.^8^ As of October 2021, there were 2085 people with intellectual disabilities and/or autism who were in-patients across England, 55% of whom had a total length of stay of over 2 years.^9^ NHS Digital statistics from September 2020 reported that the average length of in-patient stay for people with intellectual disabilities and/or autism is 5.6 years.

The Department of Health led a public consultation exercise, ‘No Voice Unheard, No Right Ignored’,^3^ which highlighted concerns that prolonged admissions under the MHA for people with intellectual disabilities without underlying mental illness does not provide therapeutic benefit, removes them from their preferred environment, estranges them from friends and family and increases their risk of exposure to restrictive practices.

This scoping questionnaire aims to explore the perspectives of healthcare workers from multiple disciplines, who work with people with intellectual disabilities and/or autism in community and in-patient settings. This group of professionals are well-placed to understand the issues and challenges facing services, patients and their families. The views of such professionals may help to explore both positive and potentially unintended negative effects of the proposed MHA reforms.

## Method

### Setting and target participants

The scoping questionnaire was aimed at all multidisciplinary team professionals working in specialist intellectual disability community and in-patient teams (intellectual disability is also known as learning disability in UK health services) in Hertfordshire Partnership University NHS Foundation Trust.

### Ethical considerations

The intended aim of the scoping questionnaire was to garner local opinions about recent changes to the legislation from the professionals working within the multidisciplinary teams. The responses are not considered to be representative of the wider national population of professionals working in this field.

The project's remit fell outside of the governance arrangements of National Health Service (NHS) research committees, and it was registered as such with the host NHS organisation. All respondents had been informed of the reasons for the questionnaire and that all data would be anonymised before sharing outside of their clinical team. Consent was implied by completion. No identifiable data about respondents has been shared outside of the Trust.

The authors assert that all procedures contributing to this work comply with the ethical standards of the relevant national and institutional committees on human experimentation and with the Helsinki Declaration of 1975, as revised in 2008.

### Data protection

Responses were anonymous and filled out electronically by following an email link.

### Questionnaire design

As a template for designing the questionnaire, we used the questions put forward in the government's consultation of the white paper for MHA reforms, adjusting where appropriate.^10^ It was created with Survey Monkey (Momentive Inc., San Mateo, CA, USA, https://www.surveymonkey.co.uk) and comprised 14 questions that included free-text, multiple choice and five-point Likert scale responses (strongly agree, agree, not sure, disagree and strongly disagree). The full questionnaire is available in the Supplementary Material available at https://doi.org/10.1192/bjo.2022.604.

### Questionnaire distribution

The questionnaires were sent via Trust internal email to all sites and departments involved in the care of people with intellectual disabilities. Responders accessed the questionnaire via electronic links and completed it online. Responses were collected between April and July 2021 (inclusive). Reminder emails were sent to all site emails periodically.

### Analysis

Raw data-sets of responses were collected and transferred to Microsoft Excel version 2202 for Windows. Primarily quantitative data were produced. Free-text answers were analysed separately by all authors. Four authors are consultant specialists working with people with intellectual disabilities, and have an in-depth knowledge of the MHA as applied to this patient population. These analyses were used to produce the qualitative findings and guide discussion. Selected quotations from responder comments have been included where relevant and have only been edited for basic grammar and spelling, without changing the content or message of the original text.

## Results

### Response rate and demographics

A total of 45 people responded, and 42 responders answered all questions. The response rate was 12.5%. Two responders did not answer the question asking about their profession and one responder did not answer one of the questions. Nine responders gave more detailed text responses in the comments sections provided.

**Fig. 1:**
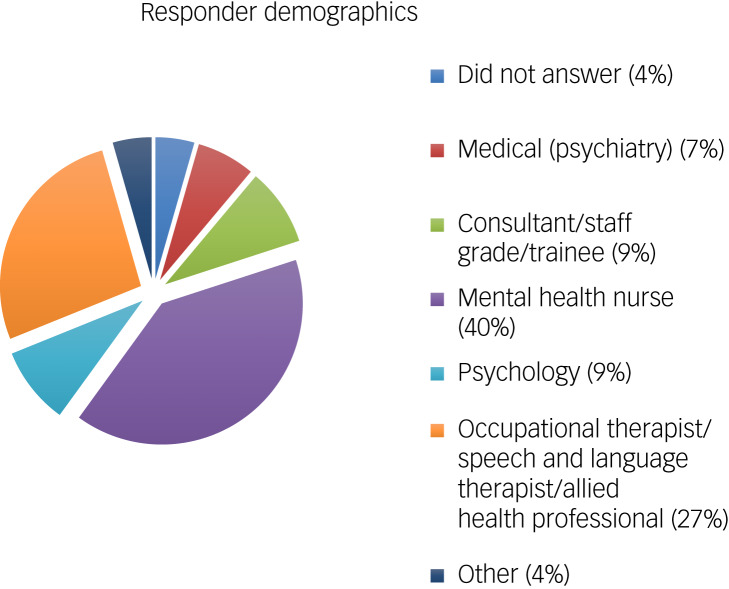
Responses: please specify your profession.

In total, 24 (53%) responders worked in an in-patient intellectual disabilities setting and 21 (47%) worked in community intellectual disability services. A total of 16% of responders were medical staff working either as psychiatric consultants, staff grade doctors or trainees. The majority of responders (80%) were non-medical professionals forming the multidisciplinary team: mental health nurses (40%), psychologists (9%), occupational therapists/speech and language therapists/allied health professionals (27%), and other (4%). Of the two people who responded ‘other’, one was a student psychiatric nurse and one was a support worker (Fig. 1).

### General views on MHA reforms (questions 1, 2, 3 and 5)

In response to the first question, 89% of responders either strongly agreed or agreed with the statement that the ‘…Government proposes changing the detention criteria so that detention must provide a therapeutic benefit to the individual’. This indicates that the majority of responders felt that the reforms were being created with the right intentions, and this is reflected in some of the comments, with one respondent stating that they ‘think these are some very positive steps forward to continue working forward to provide more enablement and empowerment to people with learning disabilities’. Another responder commented that they ‘feel that this change in legislation will reduce some of the unnecessary admissions to the assessment and treatment units for behaviours that are longstanding and are not due to mental illness’.

In response to question 2, ‘Do you agree or disagree with the proposed reform to not consider autism or learning disability to be mental disorders warranting compulsory treatment under Section 3?’, 66% of responders either agreed or strongly agreed. In response to question 3, ‘Do you agree or disagree with the proposed reforms that for patients under Section 2 where the behaviour is not considered due to an underlying mental disorder detention under Section 2 will no longer be justified?’, 60% of responders either agreed or strongly agreed. However, in response to question 5, there was a 28 *v*. 51% (strongly agree/agree versus strongly disagree/disagree) split when asked whether the reforms were justified in situations where there was no underlying mental health disorder driving behaviour, but there is a risk to themselves or others. One responder commented that ‘some people may be displaying behaviours which place themselves and others at significant risk of harm. It is concerning that this is not being looked at within the [white] paper’.

### Potential for unintended consequences

**Fig. 2:**
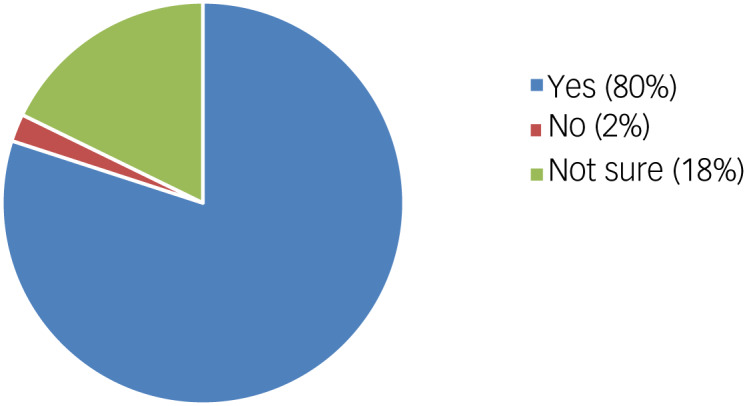
Responses: do you expect that there will be unintended consequences of the proposals to reform the way the Mental Health Act applies to people with a learning disability and people with autism?

When asked whether they thought there would be unintended consequences of the proposals to reform the way the MHA applies to people with intellectual disabilities and/or autism, 80% of responders answered ‘yes’ (Fig. 2).

### Community provisions and safety (questions 6, 7 and 11)

When asked whether the new reforms provide adequate safeguards for people with intellectual disabilities and/or autism without an underlying mental illness, 47% of responders either strongly disagreed or disagreed whereas 28% strongly agreed or agreed.

The context of this concern was touched upon by one responder, who commented:

‘ … these groups (particularly people with autism without learning disabilities) often fall between mainstream and specialist services and face difficulties having their needs met. In many instances where risk to self/others is high and imminent, use of the said provision [current wording of MHA Code of Practice] has been the only way to ensure in-patient admission to address these issues - particularly where the person has a history of refusal or poor engagement to address them in a less restrictive way.’

**Fig. 3:**
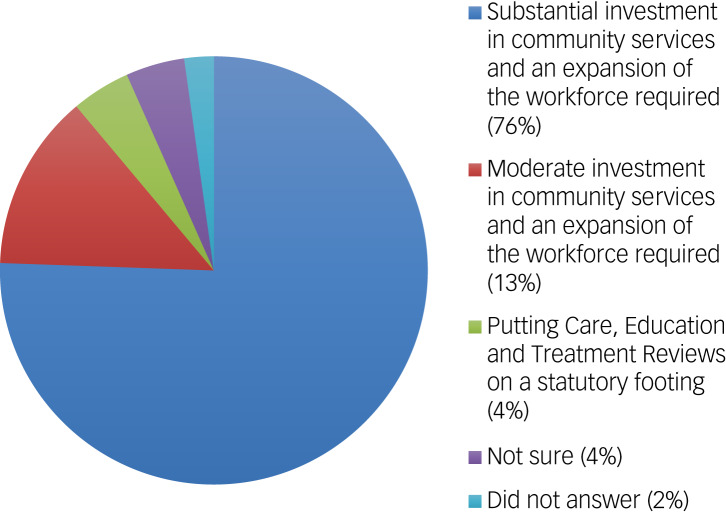
Responses: the proposed changes to the way learning disability and autism are treated in the Mental Health Act will require changes in services. Which of the following statements do you believe needs to be undertaken in your services?

A total of 40% of responders either strongly disagreed or disagreed, compared with 31% who either strongly agreed or agreed, when asked if patients who are detained under Section 2 for assessment, but whose behaviour is not considered to be caused by a mental health condition, could be managed safely in the community; 29% indicated that they were ‘not sure’. Most responders (76%) thought that ‘substantial investment in community services and an expansion of the workforce is required in advance of the changes coming into place’ (Fig. 3). It was interesting to note that this finding was consistent between responders from in-patient (83%) and out-patient (67%) teams.

Five out of nine responders who left free-text comments mentioned that there need to be improvements in community support in some way. Specific comments regarding community provision included: ‘more residential homes that can support people displaying behaviour that challenges others’, ‘the lack of robust skilled care providers’, ‘the lack of suitable robust community placements and support services’, ‘need for alternatives to admission such as respite’, ‘social care will be overwhelmed, under experienced and under-staffed’ and ‘community teams will need more staff [and] more access to psychology’.

### Inadequate time to assess for underlying mental disorder (question 4)

There was a 49 *v*. 40% (strongly agree/agree versus strongly disagree/disagree) split when asked about whether 28 days is enough time to ascertain if there is an underlying mental health condition driving the behaviour. One responder summarised the concerns surrounding assessment time in this patient group:

‘There is diagnostic overshadowing which can occur whereby people with autism and/or a learning disability are not diagnosed with a mental health condition. Twenty-eight days to assess such and try to ascertain if this is the cause is not long enough to do a thorough evidence-based assessment of the behaviour or health conditions of a person. People will be put at risk or others will be at risk of harm should this proposal occur without due thought to those with complex needs.’

### Criminal justice system (questions 9 and 10)

Responders were asked if they agreed or disagreed with the proposal that MHA reforms should only affect patients detained under the civil sections of the Act and not those in the criminal justice system: 38% strongly agreed or agreed, 35% strongly disagreed or disagreed and 27% were ‘not sure’. Further, 55% of responders thought that there would be unintended consequences for the criminal justice system because of the way the MHA reforms apply to people with intellectual disabilities and/or autism, compared with 7% who did not think this and 38% who responded that they were ‘not sure’. One responder felt that the ‘legislation change will increase reporting to the criminal justice system’.

### CETRs

Responders were also asked if they agreed or disagreed with the new proposed statutory requirement that the responsible clinician considers the findings and recommendations of CETRs in a patient's care and treatment plan, and that any deviations from this need to be explained and justified. The responsible clinician is usually the consultant psychiatrist in charge of the person's care. The majority of responders (78%) either strongly agreed or agreed, compared with only 4% who either strongly disagreed or disagreed.

## Discussion

The aim of this scoping questionnaire was to gather the perspectives of healthcare professionals who work with people with intellectual disabilities and autism on the proposed MHA reforms in England and Wales. This has enabled the exploration of both intended and potentially unintended consequences of the proposed MHA reforms. Cultural perspectives and practices can vary significantly between different professional groups, and the perspectives of each group may not necessarily be represented equally in the process of more wide-scale consultation exercises carried out by government bodies. Therefore, it was essential that this questionnaire successfully captured the views of the broad range of healthcare professionals that work within the multidisciplinary team of care providers. Both the quantitative data and the free-text responses have yielded important insights that can generate valuable discussion going forward.

**Fig. 4:**
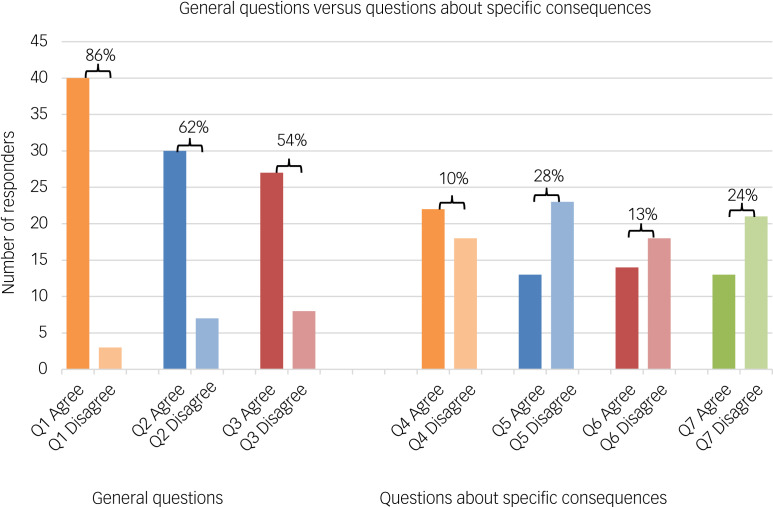
The difference in percentage split (agree versus disagree) when comparing general questions about the proposed reforms (questions 1–3) with specific questions about potential consequences of proposed reforms (questions 4–7). Opinions were more divided in the latter category.

The majority of people polled (89%) felt that the proposed reforms were intended to ensure that detention under the MHA provides a therapeutic benefit to individuals. Most responders either agreed or strongly agreed with the proposed reforms in terms of how they would affect the use of Section 3 (treatment order) and Section 2 (assessment order) of the MHA. This indicates concerns about the current system and an openness to the idea of reforms aiming to address them. Interestingly, opinions were more split in response to questions addressing potentially unintended consequences of the reforms (Fig. 4). A possible explanation for this is that people agree with the principle of the proposed reforms, but feel that community provisions are currently inadequate for the reforms to be put in place safely. Below, we highlight some concerns that have been brought up and provide some discussion around them.

### Inadequate community provisions

The majority of responders (76%) felt that substantial investment in community services and an expansion of the workforce is required in advance of the changes coming into place. The central importance of community service provisions is best summed up by one responder, who commented that ‘the lack of robust skilled care providers [in the community] is not only the reason for most admissions, but also the reason any discharges are delayed for far longer than they should be.’

This concern has been echoed in a statement by Mencap, the intellectual disability charity:

‘It is the right thing to do to require commissioners to develop community support, but this new duty must be properly funded, not left to cash-strapped local authorities who are already struggling to fund social care [ … ] Ultimately, changes to the Mental Health Act must be backed by a cross-government strategy to deliver on government promises to get people with a learning disability and/or autism out of in-patient mental health units. The government also needs to commit funding to develop the right housing and social care support to truly transform care … ’^11^

The drive to move care from in-patient beds to the community pre-dates the proposed MHA reforms. Professionals in the field have previously raised concerns regarding patient care and safety in the drive to close in-patient beds without first having adequately levelled-up community service provision.^12^ The failure of the Transforming Care Programme suggests deep-rooted problems with community provision, the complexities of which may have been underestimated, according to a 2015 report by the National Audit Office.^13^ Continued pressure to discharge in-patients despite failure to provide adequate investment in community services has raised concerns that the programme is ideologically driven, and is having a disproportionate effect on the care and safety of people with intellectual disabilities.^14^

Thus far, in-patient admission under the MHA has been a fall-back option in situations where the risk to self or others is high. Where no underlying mental health condition has been found after the 28-day assessment period under Section 2 of the MHA, the proposed reforms would remove the option of a further period of in-patient admission for treatment under Section 3 of the MHA even if there is a continued risk to self or others. This would increase reliance on community services, which may not be adequately resourced to manage a certain level of risk, potentially causing serious safety risks for a proportion of people with intellectual disabilities and/or autism and their carers/families.

### Inadequate safeguards

It is worth thinking about cases where the risk to self or others is high and attempts to manage the same risks in a community setting within the scope of current community resources have not been successful. It is difficult to imagine any clinician feeling that discharge back to the community would be in the best interests of the patient, carers or public. If an underlying mental illness driving the challenging behaviour has not been found within the 28-day period of assessment under Section 2 of the MHA, then there remain very limited options of how to proceed.

One potential consequence of the MHA reforms in situations such as these would be resorting to the use of the Mental Capacity Act (MCA) 2005 as a legal framework for managing the person as an in-patient until appropriate community placements can be found or the risk to self or others reduces. This, of course, would only apply if the patient lacks capacity, a finding that may not be applicable to many who are currently undergoing treatment. Further, patients who are in hospital under the MCA will lose the safeguards present in the MHA, such as independent tribunal, oversight of medication use by an independent second-opinion doctor and the rights of the nearest relative to request discharge. Furthermore, patients discharged after a period of treatment under Section 3 of the MHA have the right to Section 117 aftercare provisions, which are not available under the MCA. Failings in the use of the MHA have been well-highlighted in Sir Simon Wessely's independent review.^1^ However, it is important to highlight that if this potentially unintended consequence is borne out, and the use of the MCA increases, it may leave in-patients with intellectual disabilities and/or autism and their carers in a position where there are fewer safeguards in place to protect their best interests. As it stands, the MCA cannot be used as a framework to manage risk to others. This should be considered in the ongoing reviews of the MCA and the new legal framework for Liberty of Protection Safeguards.

### Lack of assessment time to recognise underlying mental health disorder

When asked if 28 days is enough time to ascertain if there is an underlying mental health condition driving the behaviour, the response was divided, at 49 *v*. 40% (strongly agree/agree versus strongly disagree/disagree). As described in the Results section, one responder mentioned diagnostic overshadowing. This specific risk has also been mentioned as part of the government's published response to the consultations on the MHA reforms.^15^

The concept of diagnostic overshadowing in people with intellectual disabilities and/or autism is well documented. Although the prevalence of mental illness is higher in people with intellectual disabilities, they are less likely to be diagnosed with a psychiatric disorder.^16^ Identification and assessment of mental health problems in people with intellectual disabilities requires consideration of a complex interplay of factors, including developmental level, neurological or sensory needs, medication side-effects, communication style, social circumstances and setting/environment. This necessitates a multidisciplinary approach, which may take a longer period of time compared with the assessment of individuals without intellectual disabilities, for whom the 28-day period of assessment under Section 2 of the MHA is considered sufficient. The proposed MHA reforms would mean that if the underlying cause of the challenging behaviour is not clearly established after 28 days, there would be no provisions to allow for a further period of monitoring. This may result in either rushed diagnoses that are not accurate (overdiagnosis), or patients being discharged prematurely despite there being an underlying mental illness that is treatable (underdiagnosis). In the latter scenario, there is the further concern of which legal framework will be used to manage the ongoing risks in the community.

### Effects associated with criminal justice system

A total of 56% of responders thought that there would be unintended consequences on the criminal justice system because of the way the MHA reforms apply to people with intellectual disabilities and/or autism. One consideration that emerged from the comments was that the proposed reforms could result in an increase in the use of forensic sections. This may occur if there are no other legal means to provide care and manage risk in the face of challenging behaviour without an underlying mental illness. People with intellectual disabilities can sometimes present with challenging behaviours, such as physical assault or destruction of property. At present, the risks associated with these behaviours can be managed under civil sections of the MHA in an in-patient setting, where efforts can be made to identify and address possible contributing factors. Although this may not be considered an ideal way to manage these risks, as described above, community provisions may not be adequately resourced to do so. Under the proposed reforms, where no underlying mental health condition has been found after the 28-day assessment period under Section 2 of the MHA, there would be no option of a further period of in-patient admission for treatment under Section 3 of the MHA, even if the person continues to present with the same challenging behaviour. This may necessitate the use of the criminal justice system and forensic sections in situations where such behaviour continues in community settings despite efforts to identify and remove triggering factors. Similar concerns have been raised in relation to the 1992 Mental Health (Compulsory Assessment and Treatment) Act in New Zealand, which legislated for the intentional exclusion of people with intellectual disabilities and no coexisting mental illness.^17^

The Royal College of Nursing's response to the government's consultation on the reforms touches on this issue by suggesting that ‘the MHA must promote the continued investment in hospital, custody and court liaison and diversion models, with the aim for these to become statutory in every locality’.^18^ It is important to consider the potential negative effects of increased reporting into the criminal justice system. Forensic sections impose more restrictions than civil sections, and patients detained under forensic sections tend to have longer in-patient admissions in more restrictive environments. For a proportion of people with intellectual disabilities and/or autism, an increase in the use of the criminal justice system may result in longer and more restrictive in-patient stays to manage challenging behaviour that may have otherwise been managed successfully with civil sections and subsequent re-integration into the community.

### CETRs

The proposed reforms would make it a statutory requirement that the responsible clinician, usually the consultant psychiatrist in charge of the patient's care, considers the findings and recommendations of CETRs in a patient's care and treatment plan. Any deviations from this will then need to be explained and justified. The majority of respondents either strongly agreed or agreed with these proposals.

The intended aim of CETRs is to help improve quality of care, safety of care and future care planning. Although the intentions of CETRs are good, in practice, their ability to make decisions that ensure the best, evidenced-based patient care can be influenced by a variety of factors, such as the experience and training of the panel members and their familiarity with the patients.

The proposed MHA reforms would increase the power and influence of CETRs. Therefore, it would be important to give due consideration to the training given to panel members to ensure an up-to-date working knowledge of current legal framework and evidence base for practice.

### Strengths and limitations

One limitation of this study is that there was a low response rate, and this is largely because of the method of distribution. To ensure all multidisciplinary professionals in the intellectual disability and/or autism teams were targeted, and to capture as many responses as possible, we decided to distribute the questionnaire by using site-specific generic staff emailing lists. However, considering the large number of people emailed, this resulted in a low response rate. Another limitation of the study is that it was conducted across one trust in South-East England, and therefore views captured may not necessarily be representative of the national population or other service providers.

The method of recruitment may well result in a tendency to capture only entrenched views on either side. However, one of the aims of this study was to capture the extent of such views and further explore potential unintended consequences of legislative change.

A key strength of the study is that we received responses from a broad range of healthcare professionals across community and in-patient multidisciplinary teams. However, a limited proportion of professionals sampled had a formal role under the MHA, and therefore may have less familiarity with the technicalities and nuances of the relevant law. This may have resulted in reduced responses from those less familiar with MHA law. To help mitigate against this, both a short section summarising the proposed reforms and a link to the government website with further information was provided with the scoping questionnaire. It is also important to acknowledge that, because of the breadth of professions sampled, some of the terms in the questionnaire, such as ‘therapeutic benefit’, may be understood differently by different professionals.

In conclusion, the findings of this scoping questionnaire provided key insights that need to be explored further and addressed before the MHA reforms are implemented. A major concern is the potential risk to people with intellectual disabilities and/or autism and to the public, where community provisions are inadequate to manage challenging behaviour that is not driven by an underlying mental illness. The potential effects on service delivery and care pathways for people with intellectual disabilities and/or autism need to be reflected in the training and education provided by NHS Trusts that are involved in the care of this patient population.

## Data Availability

The data that support the findings of this study are available from the corresponding author, B.V., upon reasonable request.

## Supplementary material

For supplementary material accompanying this paper visit http://doi.org/10.1192/bjo.2022.604. click here to view supplementary material
