# Supplementary material for: Implementing proposed reforms of the Mental Health Act for people with intellectual disability and autism: the perspective of multidisciplinary professionals in intellectual disability teams
Source: BJPsych Open. 2022 Nov 14;8(6):e197. doi: 10.1192/bjo.2022.604 (PMC9707503; doi:10.1192/bjo.2022.604)
Supplement: Supplementary file 1 [file S2056472422006044sup001.docx]

**MHA Review England and Wales 2021**

**Proposed reforms for People with a learning disability and autistic people**

**Scoping Questionnaire**

1. The Government proposes changing the detention criteria so that detention must provide a therapeutic benefit to the individual. Do you agree or disagree with this proposal?

Strongly agree

Agree

Disagree

Strongly Disagree

Not sure

Any comments…

1. Do you agree or disagree with the proposed reform to not consider autism or a learning disability to be mental disorders warranting compulsory treatment under section 3?

Strongly agree

Agree

Disagree

Strongly Disagree

Not sure

Any comments…

1. Do you agree or disagree with the proposed reforms that for patients under Section 2 where the behaviour is not considered due to an underlying mental disorder detention under Section 2 will no longer be justified?

Strongly agree

Agree

Disagree

Strongly Disagree

Not sure

Any comments…

1. Do you agree or disagree that 28 days is enough time to ascertain if there is an underlying mental health condition driving the behaviour?

Strongly agree

Agree

Disagree

Strongly Disagree

Not sure

Any comments…

1. Do you agree or disagree with the proposed reform that for patients where the driver of the behaviour is not due to an underlying mental health disorder detention under MHA will not be justified even if they are a risk to themselves or others?

Strongly agree

Agree

Disagree

Strongly Disagree

Not sure

Any comments…

1. Do you agree with the proposed reform for patients under Section 2 whose behaviour is not considered to be due to a mental health condition could be safely managed in the community?

Strongly agree

Agree

Disagree

Strongly Disagree

Not sure

Any comments…

1. Do you agree or disagree that the proposed reforms provide adequate safeguards for people with a learning disability and autistic people when they do not have a co-occurring mental health condition?

Strongly agree

Agree

Disagree

Strongly Disagree

Not sure

Any comments…

1. Do you expect that there would be unintended consequences (negative or positive) of the proposals to reform the way the Mental Health Act Applies to people with a learning disability and autistic people?

Yes

No

Not sure

Any comments…

1. The proposal to change the way that the MHA applies to people with a learning disability and autistic people should only affect civil patients and not those in the Criminal Justice System. Do you agree or disagree?

Strongly agree

Agree

Disagree

Strongly Disagree

Not sure

Any comments…

1. Do you expect that there would be unintended consequences (negative or positive) on the Criminal Justice System as a result of the proposals to reform the way the MHA applies to people with a learning disability and to autistic people? Please insert your response in the comment box.

Yes

No

Not sure

Please expand on your response…

1. The proposed changes to the way learning disability and autism are treated in the Mental Health Act will require changes in services. Which of the following statements do you believe needs to be undertaken in your services?

Substantial investment in community services and an expansion of the workforce is required in advance of the changes coming into place.

Moderate investment in community services and an expansion of the workforce is required in advance of the changes coming into place.

No investment in community services and an expansion of the workforce is required in advance of the changes coming into place.

Not sure

*Putting Care, (Education) and Treatment Reviews (CETRs) on a statutory footing*

1. The White Paper proposes a statutory requirement that the RC considers the findings and recommendations of CETRs in the patient's care and treatment plan. Deviations from the recommendations should be justified and explained by the RC. Do you agree or disagree?

Strongly agree

Agree

Disagree

Strongly Disagree

Not sure

Any comments…

1. In what service(s) do you practise? Choose those that apply

Adult ID Community

Adult ID Inpatient

Forensic-ID Community

Forensic-ID Inpatient

Enhanced Support/Crisis Team

Other

1. Please specify your profession:

Medical (Psychiatry)

Please specify : Consultant/SAS/Trainee ………………………………………..

Nurse

Psychology

OT/SALT/AHP

Other

Please specify……………………………………………..
